# Vascular endothelial cell-specific disruption of the *profilin1* gene leads to severe multiorgan pathology and inflammation causing mortality

**DOI:** 10.1093/pnasnexus/pgad305

**Published:** 2023-09-16

**Authors:** Abigail Allen-Gondringer, David Gau, Christopher Varghese, David Boone, Donna Stolz, Adriana Larregina, Partha Roy

**Affiliations:** Bioengineering, University of Pittsburgh, Pittsburgh, PA 15219, USA; Bioengineering, University of Pittsburgh, Pittsburgh, PA 15219, USA; Bioengineering, University of Pittsburgh, Pittsburgh, PA 15219, USA; Biomedical Informatics, University of Pittsburgh, Pittsburgh, PA 15206, USA; Cell Biology, University of Pittsburgh, Pittsburgh, PA 15261, USA; Dermatology, University of Pittsburgh, Pittsburgh, PA 15261, USA; Immunology, University of Pittsburgh, Pittsburgh, PA 15261, USA; Bioengineering, University of Pittsburgh, Pittsburgh, PA 15219, USA; Pathology, University of Pittsburgh, Pittsburgh, PA 15213, USA

**Keywords:** profilin, vascular, endothelial cells, actin, macrophage, inflammation

## Abstract

Actin-binding protein Profilin1 is an important regulator of actin cytoskeletal dynamics in cells and critical for embryonic development in higher eukaryotes. The objective of the present study was to examine the consequence of loss-of-function of Pfn1 in vascular endothelial cells (ECs) in vivo. We utilized a mouse model engineered for tamoxifen-inducible biallelic inactivation of the *Pfn1* gene selectively in EC (Pfn1^EC-KO^). Widespread deletion of EC Pfn1 in adult mice leads to severe health complications presenting overt pathologies (endothelial cell death, infarct, and fibrosis) in major organ systems and evidence for inflammatory infiltrates, ultimately compromising the survival of animals within 3 weeks of gene ablation. Mice deficient in endothelial Pfn1 exhibit selective bias toward the proinflammatory myeloid-derived population of immune cells, a finding further supported by systemic elevation of proinflammatory cytokines. We further show that triggering Pfn1 depletion not only directly upregulates proinflammatory cytokine/chemokine gene expression in EC but also potentiates the paracrine effect of EC on proinflammatory gene expression in macrophages. Consistent with these findings, we provide further evidence for increased activation of Interferon Regulatory Factor 7 (IRF7) and STAT1 in EC when depleted of Pfn1. Collectively, these findings for the first time demonstrate a prominent immunological consequence of loss of endothelial Pfn1 and an indispensable role of endothelial Pfn1 in mammalian survival unlike tolerable phenotypes of Pfn1 loss in other differentiated cell types.

**Significance Statement**

Actin cytoskeleton plays a key role in virtually all aspects of cell biology. In this study, we show that selective disruption of actin cytoskeleton regulatory gene Pfn1 in vascular endothelial cells leads to multiorgan pathology and death in mammals demonstrating an indispensable role of vascular endothelial Pfn1 for life. Loss of Pfn1 function in endothelial cells has a major proinflammatory consequence in vivo. We further propose that Pfn1 plays an important role in vascular endothelial and immune cell crosstalk.

## Introduction

Profilins (Pfn) belong to a family of small actin monomer-binding proteins that are found in all eukaryotes. Besides actin, Pfn also interacts with a host of other cellular proteins harboring poly-L-proline (PLP) domains as well as poly-phosphoinositides (PPIs). Among the various isoforms of Pfn (Pfn1, Pfn2, Pfn3, and Pfn4), Pfn1 is the only variant that is expressed in almost all mammalian cell types and much more abundantly than other variants (1–3). Pfn1 plays a key role in regulating actin polymerization and various actin-based cellular processes including cell migration and proliferation (4, 5). There is also evidence for Pfn1's involvement in the regulation of microtubule dynamics (6, 7). Therefore, Pfn1 may also serve as a molecular mediator for crosstalk between two major cytoskeletal systems in cells. Dysregulated expression and/or mutation of Pfn1 has been associated with a number of human diseases including various cancers, neurodegenerative, cardiovascular, and bone-related diseases (8–14).

Since the global absence of Pfn1 expression leads to early embryonic lethality during mammalian development (15), a number of previous studies utilized cell type–specific conditional homozygous knockout (KO) mouse models to examine Pfn1's function in vivo. These mouse model studies established Pfn1's importance in various biological contexts including chondrocyte function and cartilage development, glial cell adhesion/migration, cerebellar development, neurite outgrowth, and axonal regeneration after nerve injury (16–20). However, phenotypic consequences associated with selective loss-of-function (LOF) of Pfn1 in those specific cell types were tolerable. Unlike the tolerable phenotypes of Pfn1 in those cell types, we herein report that triggering endothelial Pfn1 gene disruption in adult mice induces severe pathological changes in major organ systems with proinflammatory consequences ultimately leading to mortality. These findings establish that endothelial Pfn1 function is indispensable for life. Although there are no currently known human diseases with loss of vascular endothelial Pfn1 expression, Pfn1 expression is elevated in vascular EC (EC) in atherosclerosis, diabetes, and clear cell renal cell carcinoma (8–10, 13). Therefore, the major impact of loss of endothelial Pfn1 on inflammatory pathways and molecular underpinnings revealed from the present study could provide mechanistic insights on how EC Pfn1 elevation may alter the immune microenvironment in those diseases.

## Results

### Widespread endothelial Pfn1 gene deletion leads to multiorgan pathology compromising animal survival

**Fig. 1.**
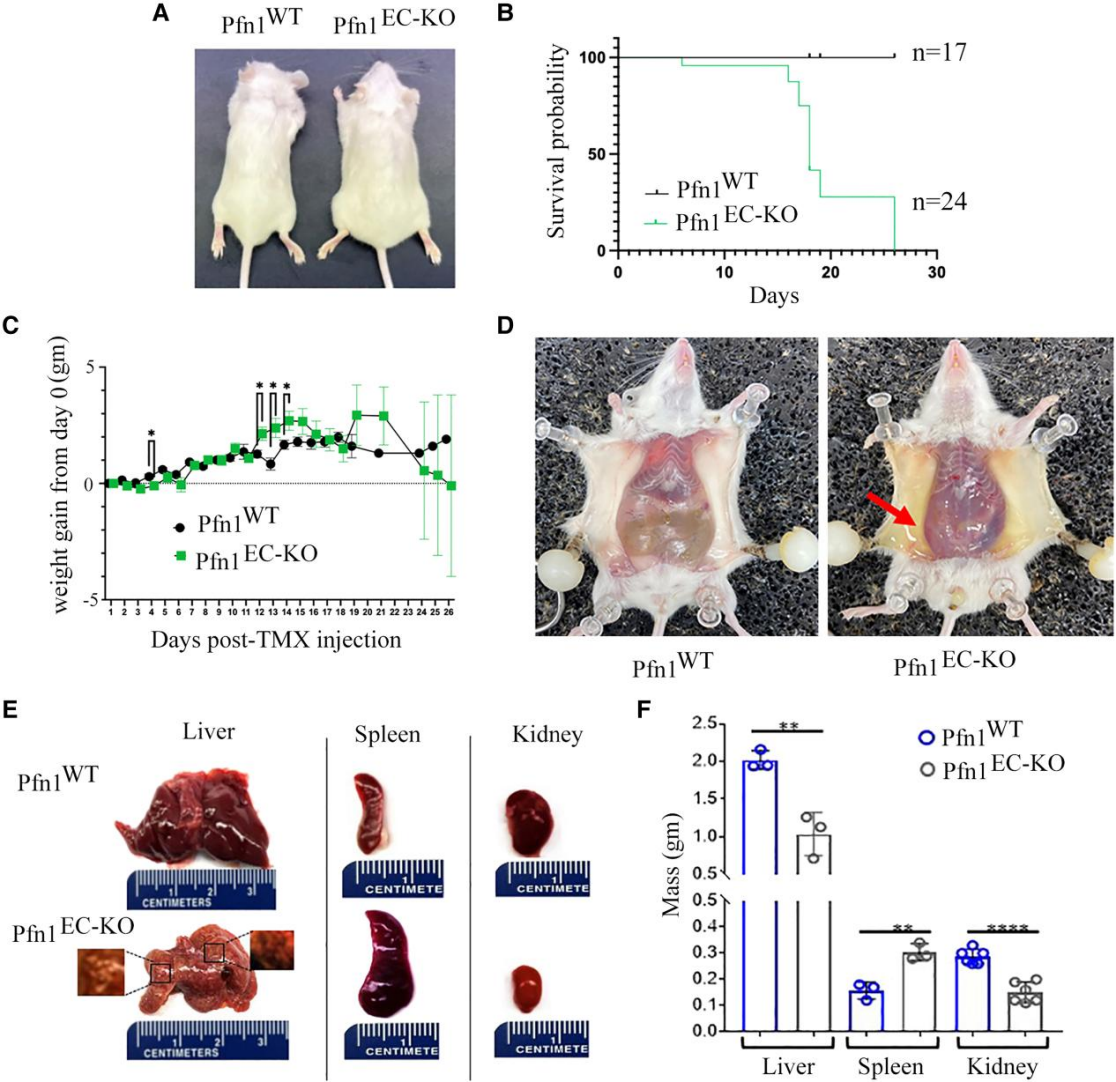
Loss of endothelial Pfn1 results in gross multiorgan pathology and compromises the survival of mice. A) Representative images of Pfn1^EC-KO^ and littermate control Pfn1^WT^ mice on d18 following the last TMX administration (denoted as d0), showing abdominal enlargement (indicative of ascites) in Pfn1^EC-KO^ mice. B) Kaplan–Meir overall survival curves of Pfn1^WT^ (*n* = 17) and Pfn1^EC-KO^ (*n* = 24) mice. C) Relative weight gain profiles of Pfn1^WT^ (*n* = 18) and Pfn1^EC-KO^ (*n* = 20) mice until the day of sacrifice. D) Necropsy of mice on d18 revealing the presence of peritoneal fluid (yellow) accumulation (arrow) in Pfn1^EC-KO^ mice. E) Left panels show representative images of the liver harvested from Pfn1^EC-KO^ and littermate control Pfn1^WT^ mice. The liver of the Pfn1^EC-KO^ mouse is smaller than the one dissected from the littermate and presents pale nodules of 2–4 mm (*left inset*), which alternate with areas of vascular congestion and subcapsular hemorrhage (*right inset*). Middle panels show an increase in size and congestion of the spleen from the Pfn1^EC-KO^ mouse compared to that from the littermate. The right panels show a reduction in the size of the kidney of the Pfn1^EC-KO^ mouse compared to that from the littermate control. F) Bar diagram illustrating the weight (means ± std. dev) of livers, spleens, and kidneys from Pfn1^EC-KO^ and littermate control Pfn1^WT^ mice (*n* = 3 mice/genotype). **P < 0.01, ***P < 0.001, and ****P < 0.0001.

A number of mouse models have been developed to date to either constitutively or inducibly modulate gene expression in all or a subset of EC (21). Among these, the Tg(Cdh5-Cre/ERT2) (Cre expressed under the control of the VE-cadherin promoter) line is one of the most widely used models to trigger Cre-mediated gene deletion in pan-EC in a tamoxifen (TMX)-inducible fashion without non-EC expression of Cre (22, 23). We adopted this transgenic line to recently engineer Pfn1^flox/flox^:Cdh5-Cre-ERT2 mice that displayed defect in developmental retinal angiogenesis upon TMX-induced excision of the Pfn1 gene (24). However, since that study was exclusively limited to phenotypic assessment of retinal vasculature in the early postnatal period (up to day P9), the long-term consequence of endothelial Pfn1 deficiency remained unclear. In this study, we triggered Pfn1 gene deletion in EC in adult Pfn1^flox/flox^:Cdh5-Cre-ERT2 mice (referred to as Pfn1^EC-KO^ from hereon) utilizing a 5-day time course injection of TMX. Littermate control Cre-negative mice (referred to as Pfn1^WT^ from hereon) were also subjected to TMX administration following the same protocol. Genotyping performed on ear-clipped tissues confirmed the expected 700-bp amplicon upon deletion of Exon 1 of the *Pfn1* gene in Cre^+^ mice (Fig. S1A). We confirmed Cre-induced downregulation of endothelial Pfn1 at both mRNA (by qRT-PCR) and protein (by immunoblot) levels in CD31-enriched cell population (a commonly employed technique to isolate EC) isolated from the kidney (a vascular rich organ) of Pfn1^EC-KO^ relative to Pfn1^WT^ animals (Fig. S1B and C). As an additional specificity control, we confirmed that Pfn1 expression was unchanged in CD31-negative cells. Our results showing ∼60% reduction of Pfn1 expression is completely consistent with previously published findings demonstrating significant animal-to-animal and organ-to-organ variation in the recombination efficiency of Cre in a Cdh5-Cre-ERT2 mouse model with average efficiency approximately equal to 60% in kidney CD31+ cells (25). General health assessment revealed that labored breathing, limited mobility, and scruffy coat were the most noticeable external features of Pfn1^EC-KO^ animals. At the necropsy gross inspection, Pfn1^EC-KO^ mice showed enlarged abdomen (Fig. 1A) with the majority of animals requiring compassionate euthanasia within the 15–18 day time window following completion of the last TMX injection (Fig. 1B). Daily weight monitoring revealed a commensurate increase in weight gain for either genotype in the initial phase, but Pfn1^EC-KO^ mice eventually exhibited markers of poor health alongside a slightly larger weight gain (Fig. 1C). Abdominal distension of Pfn1^EC-KO^ mice correlated with the presence of ascites (likely explains continued weight gain in the presence of poor health) and hemorrhage in the thorax and abdominal cavities (Fig. 1D). Compatible with portal hypertension, the spleens from Pfn1^EC-KO^ mice were enlarged whereas the livers were reduced in size with the presence of subcapsular pale-yellow nodules from 3 to 5 mm in diameter. Likewise, the kidneys of Pfn1^EC-KO^ mice were visibly smaller compared to their littermate Pfn1^WT^ controls (Fig. 1E and F). Given the dramatic impact of loss of endothelial Pfn1 on the overall health as well as organ size, we also performed an exploratory metabolic assessment of a limited set (four animals per genotype) of Pfn1^WT^ and Pfn1^EC-KO^ mice. A general trend of lower serum levels of albumin, total protein, and globulin, in Pfn1^EC-KO^ relative to Pfn1^WT^ animals is further suggestive of a negative impact of the loss of endothelial Pfn1 on liver and kidney functions (Supplementary Fig. S2). Collectively, these findings demonstrate that endothelial Pfn1 function is indispensable for normal physiology and mammalian survival.

**Fig. 2.**
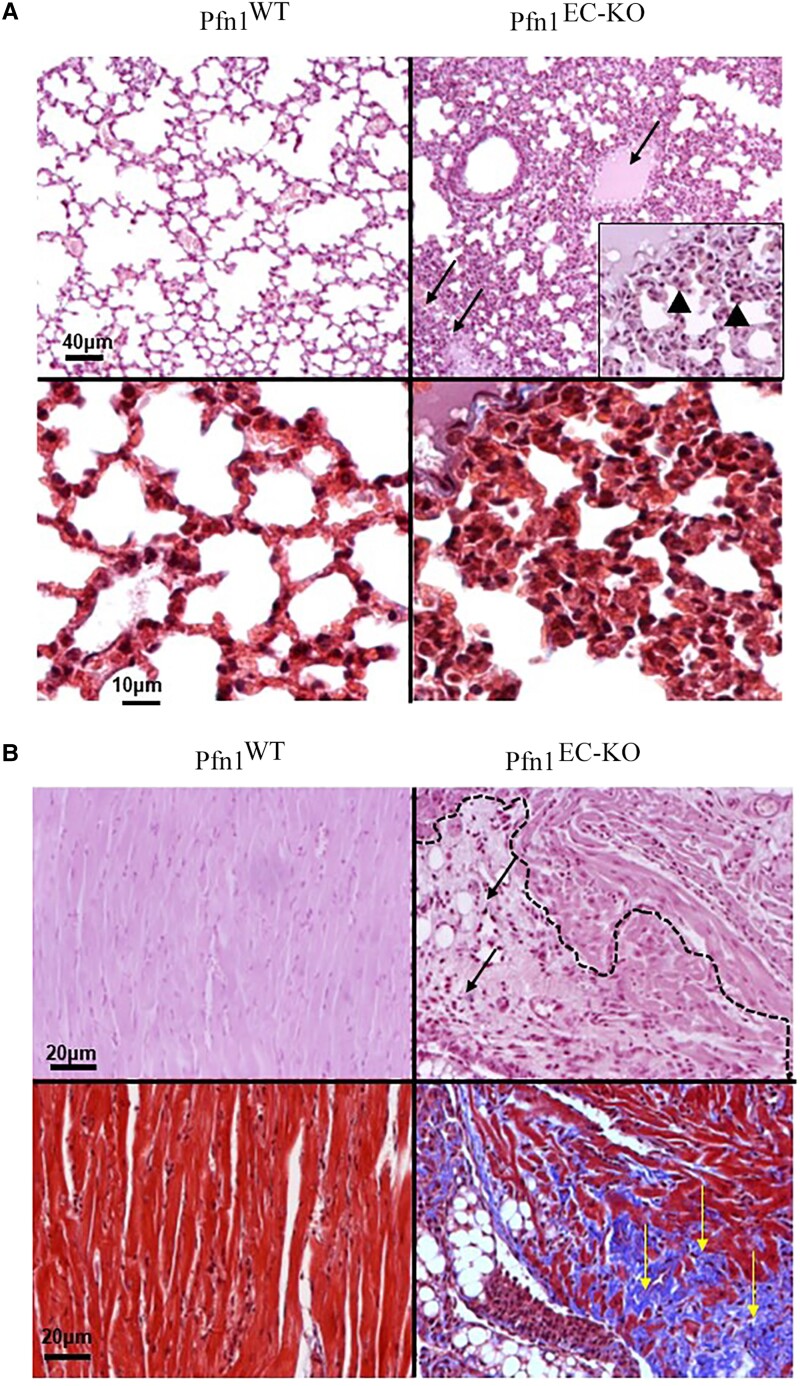
Histopathological findings in mouse lungs and hearts lacking endothelial Pfn1. A) Upper panels are representative images of H&E staining sections from the lungs of Pfn1^EC-KO^ and littermate control Pfn1^WT^ mice harvested on d18 showing decrease of the airspace, alveolar edema (arrows), extravasation of erythrocytes, and alveolar capillaries filled with blood and protruding in the alveoli (inset, arrowheads) in Pfn1^EC-KO^ lung. Lower panels are lung sections stained with Masson trichrome showing a lack of fibrosis. Upper panels H&E, ×100. Inset, ×500. Lower panels ×500. B) Upper panels are representative images of H&E staining sections of the hearts of a littermate control Pfn1^WT^ mouse (left) showing healthy heart muscle and a Pfn1^EC-KO^ mouse (right), with an area of chronic infarct (dotted line) with mononuclear cell inflammatory infiltrate (arrows). Lower panels are sections of the heart stained with Masson trichrome showing extensive fibrosis in the area of the heart infarct (arrows). Upper panels, ×200. Lower panels ×200.

**Fig. 3.**
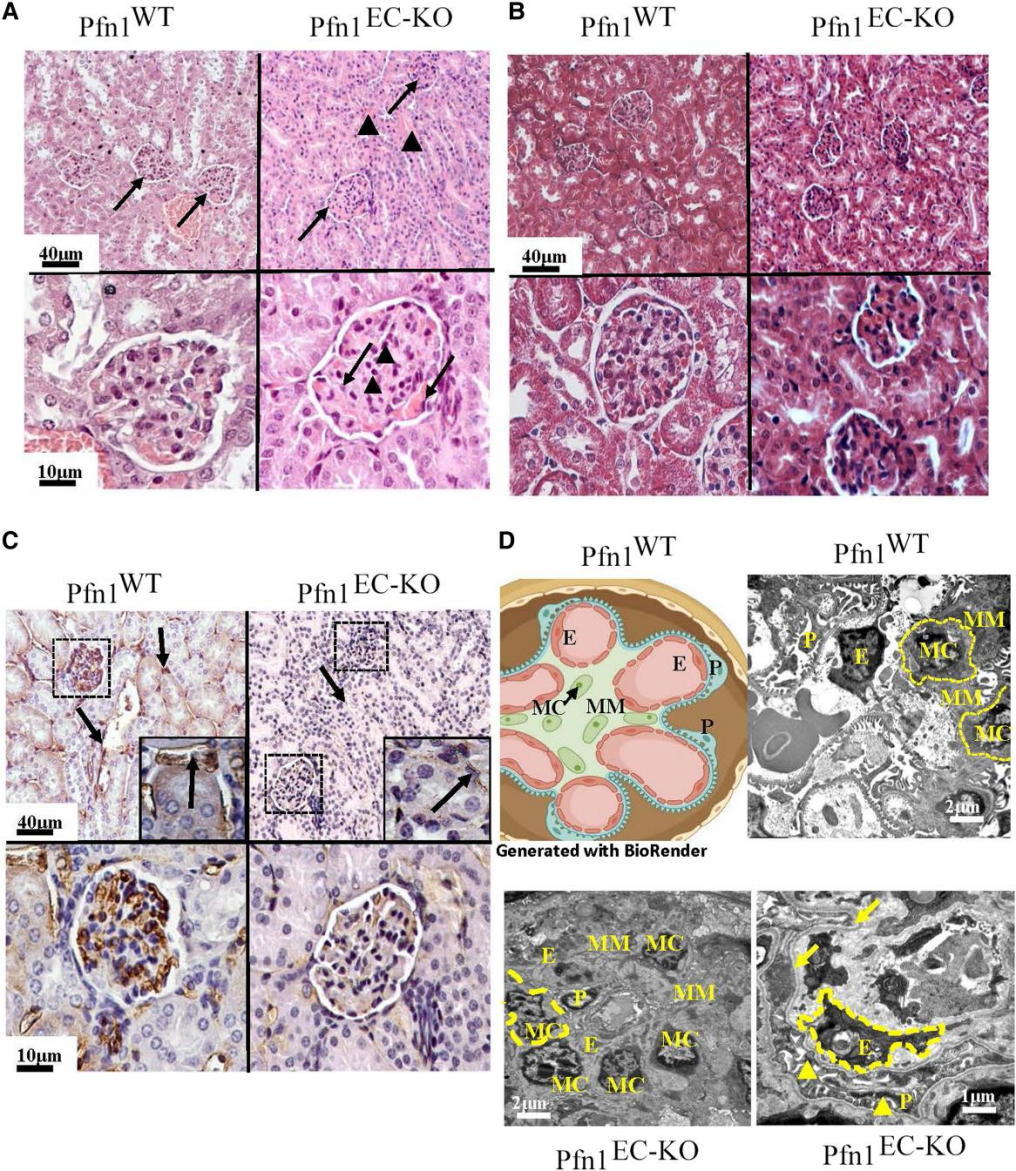
Histopathological findings in mouse kidneys lacking endothelial Pfn1. A) Upper panels are representative images of H&E staining (×100) sections of the kidneys of a littermate control Pfn1^WT^ (left) vs Pfn1^EC-KO^ mouse (right). The glomeruli of Pfn1^EC-KO^ mice were smaller than those of the littermate controls (arrows), and the tubules showed apoptotic epithelial cells, intraluminal edema, and erythrocytes (arrowheads). Lower panels are higher magnification images (×500) showing proliferation of mesangial cells (arrowheads), an abundance of eosinophilic deposits compatible with mesangial matrix, and dead EC (arrows) in Pfn1^EC-KO^ mice. B) Masson trichrome staining of kidney sections shows the absence of fibrosis (upper panels ×100, lower panels ×500). C) CD31 staining of kidney sections shows normal vascularization of glomeruli (arrows) and surrounding the tubules (arrowheads) in littermate control Pfn1^WT^ mouse (upper left panel). The upper right panel shows a lack of mature capillaries in the glomeruli (arrows) and between tubules (arrowheads) in sections of kidneys from Pfn1^EC-KO^ mice. Lower panels are high magnifications of glomeruli showing the presence of mature blood vessels in the glomerulus of a Pfn1^WT^ mouse (left) contrasting scarce endothelial cells with immature characteristics in the glomerulus of a Pfn1^EC-KO^ mouse (right). Upper panels ×100, lower panels ×500. D) Upper left: cartoon of a normal glomerulus (generated using the BioRender illustration software). The upper right panels are TEM images (×5,000) of a glomerulus from a Pfn1^WT^ mouse showing normal endothelial cells (E), podocytes (P), and mesangial cells (Mc). Lower panels are images of a glomerulus from a Pfn1^EC-KO^ mouse showing a high number of mesangial cells (arrows) and deposits of the mesangial matrix (Mm) surrounding a collapsed capillary (dotted area). The lower right shows a capillary lined by apoptotic endothelial cells, thickening of the basal membrane (asterisk), and apoptotic podocytes.

**Fig. 4.**
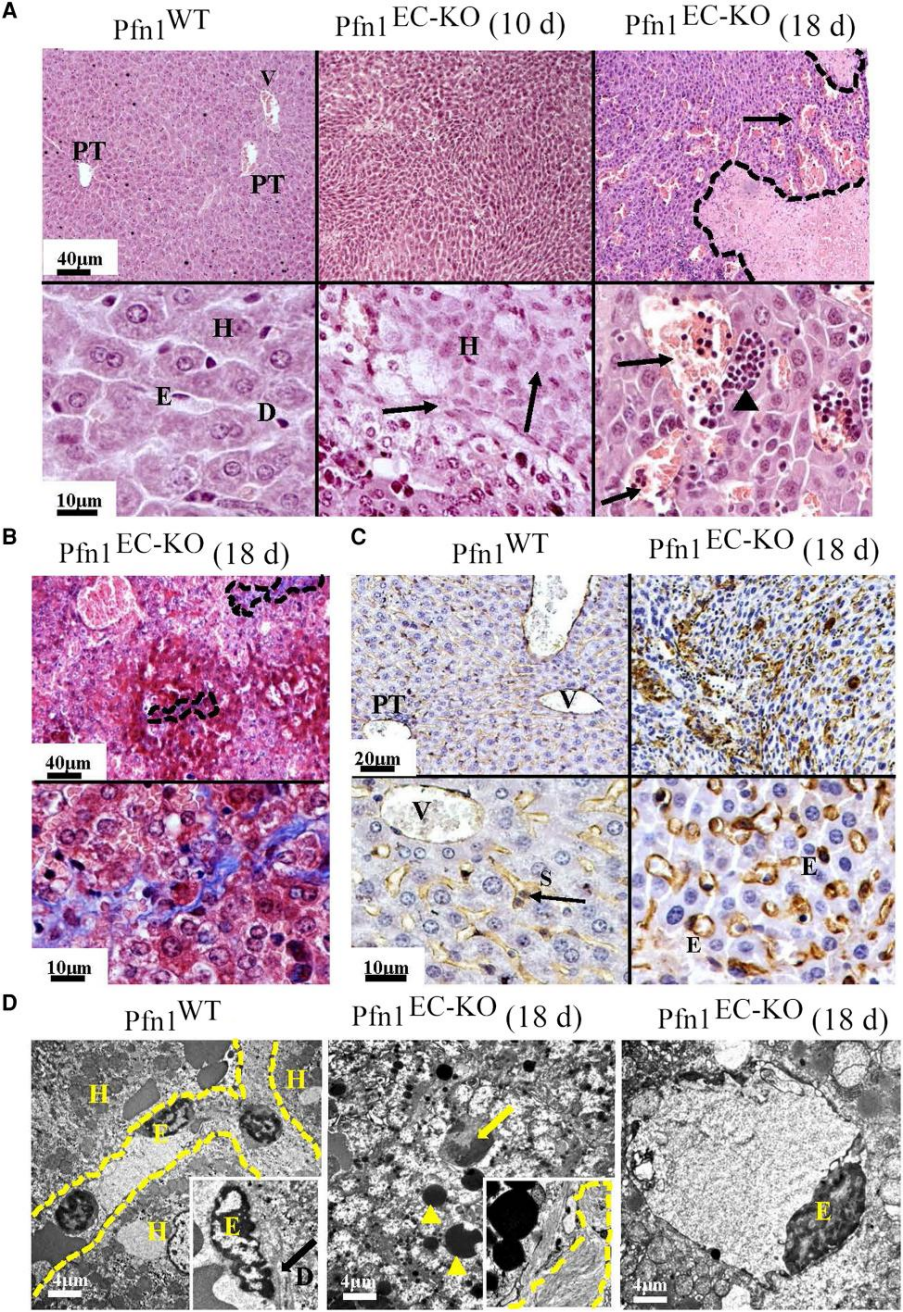
Histopathological findings in mouse livers lacking endothelial Pfn1. A) Representative images showing normal liver histology (left) or the histopathological changes observed 10 days (center) or 18 days (right) after TMX administration in Pfn1^EC-KO^ mice. Upper left is a section of the liver from a littermate control Pfn1^WT^ mouse illustrating the characteristic acinar units composed of the central vein, portal tracts, and hepatocytes arranged radiating toward the central vein. Lower left is a higher magnification of the same section showing polygonal hepatocytes arranged in sheets, the space of Disse, and sinusoids lined by EC. The upper middle is a section of the liver of a Pfn1^EC-KO^ mouse 10 days after administration of TMX showing disruption of the acinar architecture, with areas devoid of central veins. Lower middle, higher magnification shows areas with apoptotic hepatocytes, lack of spaces of Disse, and lack of sinusoids. The upper right is a section of the liver of a Pfn1^EC-KO^ mouse 18 days after administration of TMX showing ample areas of infarcts (dotted lines) with inflammatory infiltrates composed of mononuclear cells surrounded by angiectasias (arrow). Lower right, a higher magnification shows foci of extramedullar hematopoiesis surrounded by small hepatocytes, some of them lacking the polygonal shape. H&E, upper panels ×100, lower panels ×500. B) Representative sections stained with Masson trichrome of the livers in A, showing the presence of fibrosis in the liver of the Pfn1^EC-KO^ mouse. Upper panel ×100, lower panel ×500. C) Upper left, immunostaining with CD31 of a liver section from a littermate Pfn1^WT^ mouse showing the normal vascular architecture of the acini including the central vein and portal tracts. Lower left, a higher magnification shows mature branched acinar sinusoids radiated around the central vein. Upper right, immunostaining with CD31 of a liver section of a Pfn1^EC-KO^ mouse showing a disarrayed pattern that lacks the typical sinusoidal shape. The lower right panel at higher magnification shows immature blood vessels without branches and a lack of sinusoids. Upper panels ×200, lower panels ×500. D) Upper left is a TEM image of the liver from a littermate Pfn1^WT^ mouse showing a sinusoid lined by EC and surrounded by alive hepatocytes. A higher magnification (inset ×10,000) shows the nucleus of one endothelial cell, the space of Disse (arrow). The center is a TEM image of a liver from a Pfn1^EC-KO^ mouse showing necrotic hepatocytes and microvesicular fat accumulation (×5,000). The right panels show an isolated immature neovessel in the liver from a Pfn1^EC-KO^ mouse (×5,000). CV, central vein; D, Disse space; E, endothelial cells; H, hepatocyte; I, infarct; PT, portal tract; S, sinusoid.

In the histological analyses, the main pathological features of Pfn1^EC-KO^ mice were found in the heart, lung, liver, and kidney. Compared to their littermate controls, the lungs from Pfn1^EC-KO^ mice harvested on day 18 after the last TMX injection showed significant decrease of the airspaces, alveolar edema with extravasation of erythrocytes, capillaries filled with blood and endothelial cells, and pneumocytes protruding in the alveoli lumen and without evident fibrosis (Fig. 2A). The hearts from Pfn1^EC-KO^ mice showed areas of chronic infarcts with inflammatory infiltrate composed of mononuclear cells and extensive fibrosis (Fig. 2B). Histological analyses of the kidneys from Pfn1^EC-KO^ mice showed smaller glomeruli, proliferation of mesangial cells, and abundant deposit of mesangial matrix (Fig. 3A) with no evidence for fibrosis (Fig. 3B). CD31 immunostaining revealed a lack of capillaries in the cortical areas of the kidneys in Pfn1^EC-KO^ mice; the glomeruli of Pfn1^EC-KO^ kidneys also showed a significant decrease in capillaries, and the scarce capillaries present had the characteristics of immature neovessels (Fig. 3C). Transmission electron microscopy (TEM) analyses showed apoptosis of endothelial cells and podocytes and a collapse of the urinary filtration space and further confirmed the increased number of mesangial cells and thickening of basal membranes observed in the sections stained with hematoxylin and eosin (H&E) staining (Fig. 3D). In the liver, the histopathological changes were observed 10 days after the last TMX administration, which progressed significantly by day 18. On day 10, Pfn1^EC-KO^ livers showed distorted acinar architecture and collapsed sinusoids lacking EC lining and disappearance of the Disse spaces. The hepatocytes were small, and most of them presented cytoplasmic fat vesicles. There were areas of necrosis with inflammatory cellular infiltrates composed of mononuclear cells (Fig. 4A). On day 18, Pfn1^EC-KO^ livers showed extensive subcapsular infarcts surrounded by fibrosis, angiectasia, and abundant foci of extramedullary hematopoiesis (Fig. 4A and B). There was a general defect in sinusoid development with several CD31-positive cells distributed throughout the hepatic parenchyma in a disorganized pattern and some of them with characteristics of immature vessels (Fig. 4C). TEM analysis showed the presence of necrotic hepatocytes with cytoplasmic fat vesicles, extensive deposits of collagen, and isolated immature neovessels (Fig. 4D). TUNEL staining further confirmed extensive apoptosis throughout the liver in Pfn1^EC-KO^ mice (Supplementary Fig. S3). Together, these microscopic and histological findings in Pfn1^EC-KO^ mice demonstrate a severe defect in the development, expansion, and maturation of blood vessels. The expected defective blood circulation is consistent with pathological features including infarcts, portal hypertension, neovessel formation, and inflammatory infiltrates, likely contributing to impaired liver and kidney function.

### Endothelial loss of Pfn1 has proinflammatory consequences

**Fig. 5.**
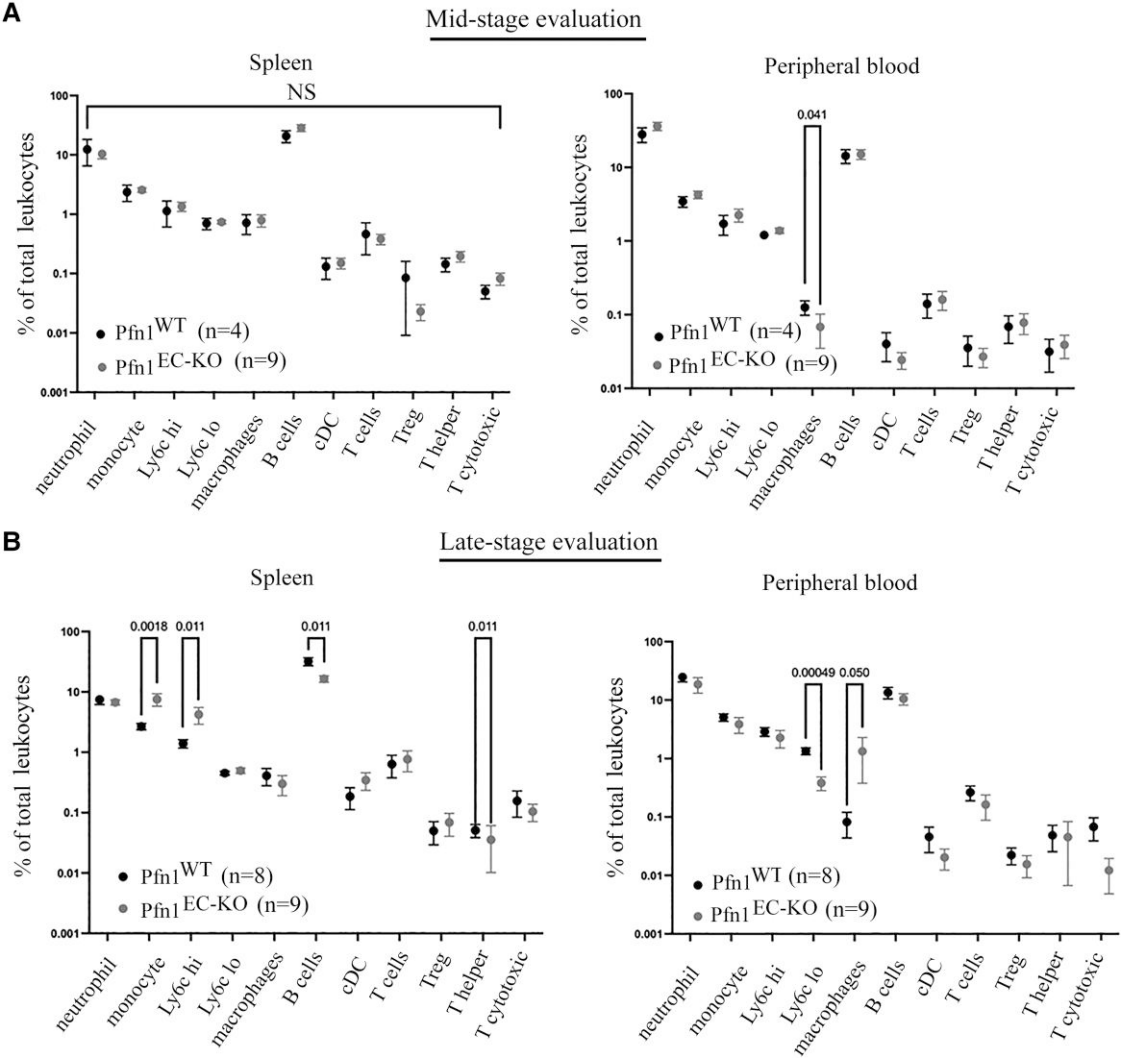
Effect of endothelial Pfn1 depletion on immunological profile in vivo. Flow cytometry–based quantification of various subtypes of immune cells expressed as a % of total leukocytes in either spleen or peripheral blood of Pfn1^WT^ vs Pfn1^EC-KO^ mice either 8–10 days (mid-stage, *panel A*) or 15–18 days (late-stage, *panel B*) after last TMX administration. Data representative of at least three separate litters with “*n*” representing the total number of mice in each group pooled from different litters. The “*P*” values, when significant (or close to being significant), are indicated above the comparison bar.

Next, to determine whether there is any global immunological impact of endothelial Pfn1 ablation in vivo, we next performed comprehensive analyses of various immune cell subtype population in peripheral blood and spleen harvested from mice at either the mid-stage (10–11 days after last TMX injection) or the late-stage (18 days after last TMX injection) of Pfn1-depleted condition. Mid-stage immune profiling showed evidence for a dramatically (as high as ∼10-fold) elevated total leukocytes, various lymphocytes, and monocyte population in the peripheral blood (but not in the spleen) of Pfn1^EC-KO^ relative to Pfn1^WT^ mice (Supplementary Fig. S4A and B). End-stage immune profiling revealed a significant decrease in the total splenic counts of leukocytes and various subtypes of immune cells including neutrophils, macrophages, anti-inflammatory Lys6C^lo^ monocytes, B cells, and T-helper cells (Supplementary Fig. S4C), a finding that is consistent with portal hypertension and splenic dysfunction. Although the total circulating leukocyte count normalized between the two groups at the end stage, the total counts of Lys6C^lo^ monocytes and adaptive immunity–associated cells (B cells and various T cells) showed a decline in Pfn1^EC-KO^ animals (Supplementary Fig. S4D). However, when we specifically analyzed various subtypes of immune cells as a % of total leukocytes, end-stage Pfn1^EC-KO^ animals exhibited higher relative abundance of splenic monocytes and circulating macrophages compared to Pfn1^WT^ animals in a statistically significant manner (Fig. 5). A greater proportion of proinflammatory Lys6C^hi^ with concomitant reduction of the anti-inflammatory Lys6C^lo^ pool in splenic and circulating monocytes/macrophages is further indicative of skewing of myeloid-derived subpopulation of immune cells toward a proinflammatory phenotype in Pfn1^EC-KO^ animals.

**Fig. 6.**
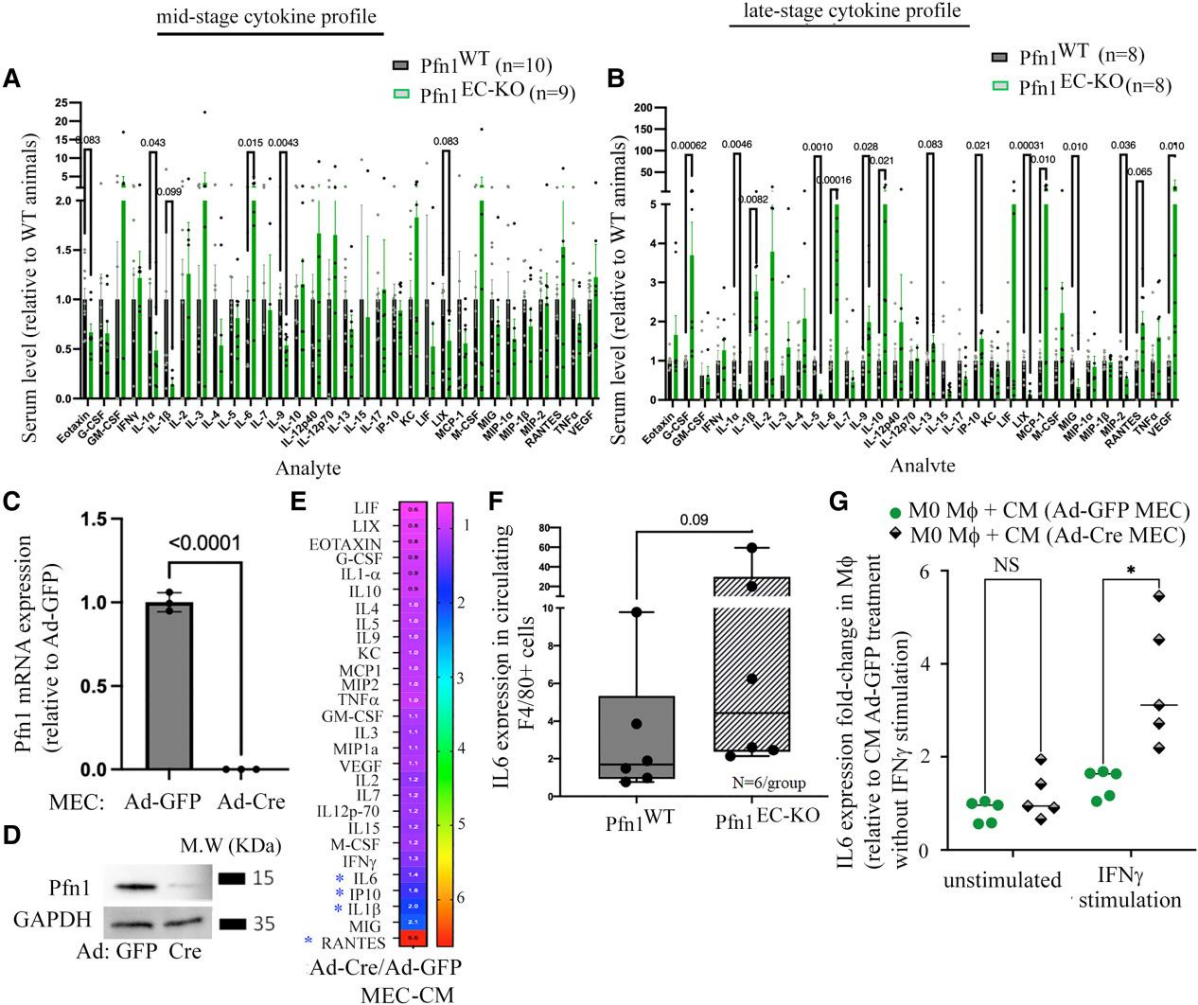
Effect of endothelial Pfn1 depletion on immunomodulatory cytokine/chemokine secretion in vitro and in vivo. A and B) Relative abundance of indicated cytokines/chemokines circulating in the serum of Pfn1^WT^ vs Pfn1^EC-KO^ mice collected either 8–10 days (mid-stage, panel A) or 15–18 days (late-stage, panel B) after the last TMX administration. Data representative of at least three separate litters with “*n*” representing the total number of mice in each group pooled from different litters. C and D) Real-time quantitative RT-PCR (*panel C*; *n* = 3 experiments) and immunoblot (*panel D*) validations of Pfn1 depletion in Ad-Cre–infected EC with Ad-GFP–infected cells serving as control (18S RT-PCR and GAPDH blots serve as the housekeeping gene control and loading control for RT-PCR and immunoblot experiments, respectively). E) Relative abundance of indicated cytokines/chemokines in the CM of Ad-Cre–infected EC relative to Ad-GFP–infected control cells (data represent the average fold change based on three independent experiments). F) Relative abundance of IL6 mRNA expression in circulating primary F4/80+ cells isolated from mice with the indicated genotypes (*n* = 6 mice/group pooled from two separate litters). G) Relative abundance of IL6 mRNA expression in macrophages differentiated from primary BMDM and exposed to the CM of Ad-Cre– vs Ad-GFP–infected EC, either in the absence or in the presence of IFNγ stimulation (data summarized from five independent experiments). *P < 0.05 and **P < 0.01.

The foregoing findings led us to query whether endothelial Pfn1 depletion causes changes in the circulating levels of immunomodulatory cytokines and chemokines in vivo. To address this question, we profiled serum of Pfn1^WT^ and Pfn1^EC-KO^ animals for a panel of 32 immunomodulatory cytokines and chemokines using a Luminex-based assay at either mid-stage or end-stage of Pfn1 depletion. Based on these analyses, mid-stage Pfn1^EC-KO^ mice showed a statistically significant 2.45-fold increase in the circulating level of proinflammatory cytokine IL6 (*P* = 0.015) relative to Pfn1^WT^ mice (Fig. 6A). Pfn1^EC-KO^ animals also displayed a general trend of increase in the circulating levels of GM-CSF, M-CSF, KC/CXCL1, and RANTES/CCL5, but these changes did not reach statistical significance due to larger animal-to-animal variation. Changes in the immunomodulatory factors were more prominent at the end-stage evaluation. In addition to continued elevation of IL6 (increased by ∼5-fold; *P* = 0.00016), circulating levels of several other factors including VEGF, G-CSF, IL1β, IL9, IL10, IP10/CXCL10, and MCP-1/CCL2 increased in statistically significant manners in end-stage Pfn1^EC-KO^ relative to Pfn1^WT^animals (Fig. 6B). A few additional cytokines including IL2, RANTES/CCL5, and LIF also exhibited a similar trend, but these changes did not reach statistical significance because of larger animal-to-animal variations. IL1α was the only circulating cytokine that appeared to show a reduction in both early and late-stage Pfn1^EC-KO^ animals relative to Pfn1^WT^ controls. We next asked whether EC could be a potential cellular source for some of these elevated proinflammatory factors as seen in Pfn1^EC-KO^ mice. To address this question, we transduced 2D cultures of our recently generated human telomerase reverse transcriptase (hTERT)–immortalized EC isolated from Pfn1^flox/flox^ FVB mice (26) with adenovirus encoding either Cre (Ad-Cre) or GFP (Ad-GFP) as control and performed Luminex analyses of the conditioned media (CM) harvested from these cells. Ad-Cre-mediated acute loss of Pfn1 expression in cultured MEC cells was confirmed by both qRT-PCR and immunoblot analyses (Fig. 6C and D). Although our in vitro experiments with cultured EC did not perfectly recapitulate the in vivo findings (this was not unexpected since immunomodulatory factors are secreted by multiple different cell types in vivo), we were able to see some degrees of qualitative concordance between our in vitro and in vivo results. For example, similar to our findings in Pfn1^EC-KO^ mice, secreted levels of IL-6, CXCL10, RANTES/CCL5, and IL1β were elevated upon Pfn1 gene KO in EC (Fig. 6E). We also detected prominent elevation of CXCL9/MIG9 in Pfn1-depleted EC, a finding that contrasted a modest reduction of this chemokine in Pfn1^EC-KO^ relative to Pfn1^WT^ mice. Overall, these results suggest that EC could at least partly contribute to elevated levels of some of the circulating proinflammatory factors as seen upon loss of Pfn1 expression in vivo.

Among the various immunomodulatory factors that we profiled, IL6 is a cytokine that was not only reproducibly elevated in both early and late stages of Pfn1^EC-KO^ mice in a statistically significant manner, but was also one of the most robustly elevated cytokines. Although our in vitro studies qualitatively recapitulated IL6 elevation in EC in response to loss of Pfn1 expression, the fold change did not commensurate with that seen in vivo. This begged a question as to whether other types of cells could further contribute to IL6 elevation in response to endothelial Pfn1 loss in vivo. Given macrophages are one of the major sources of circulating IL6 (27), we next analyzed IL6 expression in F4/80+ circulating monocytes/macrophages isolated from Pfn1^WT^ and Pfn1^EC-KO^ mice by qRT-PCR analyses. Although the *P*-value (0.09) did not reach statistical significance (because of large animal-to-animal variation), monocytes/macrophages from Pfn1^EC-KO^ mice indeed showed a trend of higher IL6 expression relative to those harvested from Pfn1^WT^ mice (Fig. 6F). To further determine whether loss of Pfn1 in EC could somehow simulate macrophages to upregulate IL6 expression, we performed indirect coculture experiments. Specifically, we exposed M0 macrophages derived from primary bone marrow–derived mouse monocytes (BMDMs) to the CM of Ad-GFP- vs Ad-Cre-transduced EC, either in the absence or in the presence of IFNγ stimulation (mimics a proinflammatory milieu). These indirect coculture experiments showed that endothelial Pfn1 depletion potentiates IFNγ-dependent IL6 induction in macrophages (Fig. 6G). These data further support a scenario that depletion of Pfn1 somehow enhances the ability of EC to crosstalk with macrophages and upregulate IL6 expression through a paracrine mechanism.

### Pfn1 depletion in EC leads to increased activation of IRF7 and STAT1

**Fig. 7.**
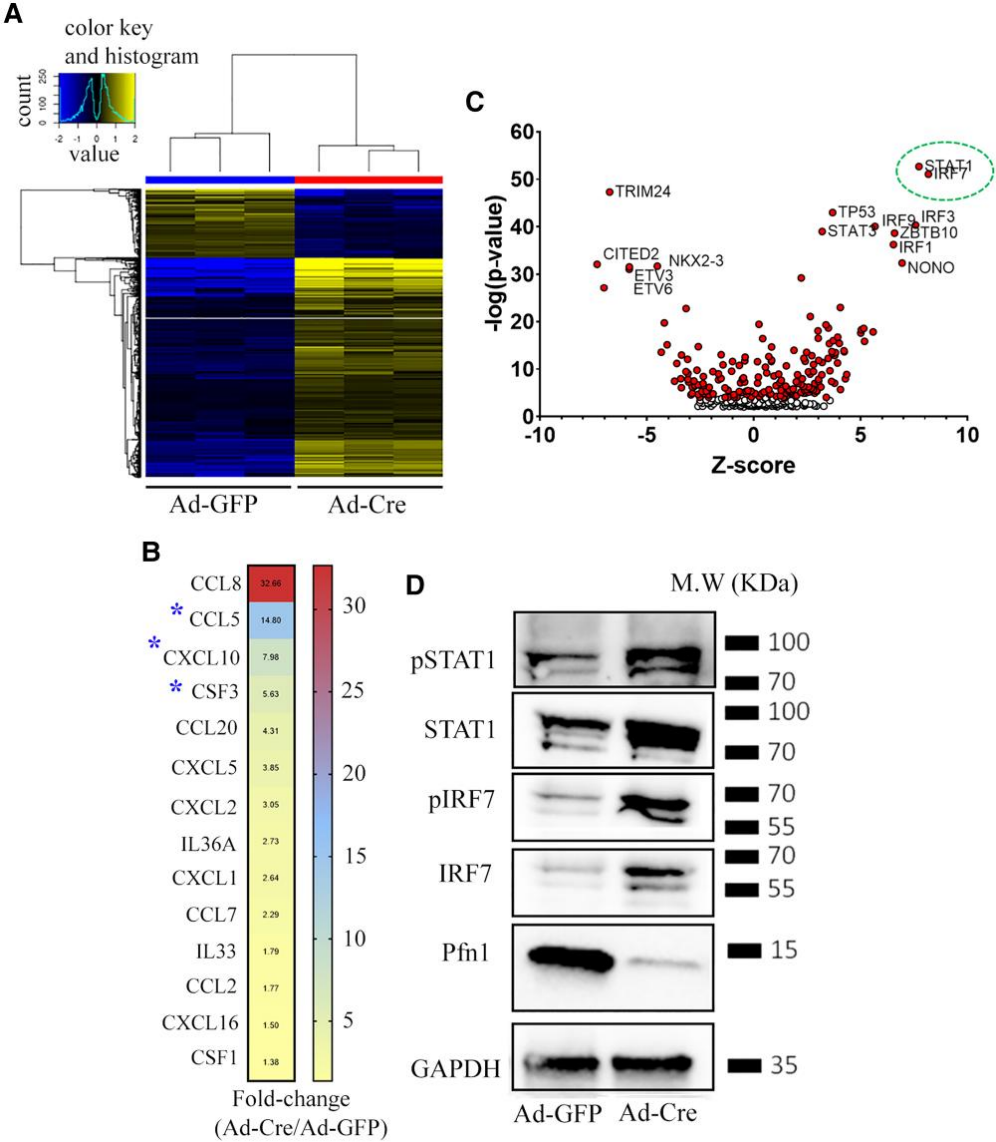
Impact of Pfn1 depletion on EC transcriptome. A) Heat plot showing differentially expressed genes between Ad-GFP– and Ad-Cre–infected EC (genes color coded by blue and yellow denote transcriptionally downregulated and upregulated genes in Ad-Cre relative to Ad-GFP–infected cells; data summarized from three biological replicates). B) Heat plot showing transcriptionally altered cytokines/chemokines as a result of Pfn1 depletion in EC (numbers alongside indicate the average fold change in Ad-Cre relative Ad-GFP groups; asterisks denote robustly altered cytokines/chemokines that are in agreement with serum and/or CM Luminex data). C) A volcano plot (*P*-value vs *Z* score) displaying IPA-predicted activated and inhibited transcriptional regulators in Ad-Cre– vs Ad-GFP–infected cells. Top IPA-predicted activated transcription factors (based on the absolute *Z* score and negative log *P*-value) are outlined by a green oval. D) Representative immunoblots showing the relative levels of the total as well as phosphorylated forms of STAT1 and IRF7 between Ad-GFP– and Ad-Cre–infected cells (GAPDH blot serves as the loading control; Pfn1 blot confirms Ad-Cre–induced suppression of Pfn1 expression).

In order to gain mechanistic insights into biological and signaling pathways that are perturbed in EC upon loss of Pfn1 expression, we performed RNA-sequencing (RNAseq) analyses to transcriptionally profile Ad-GFP- vs Ad-Cre-transduced EC in an acute setting ensuring that cell viability was not compromised. With the absolute fold change in expression and the false-discovery rate (FDR) set to >1.5 and <0.05, respectively, we found a total of 1,035 differentially expressed genes (DEGs—789 upregulated and 246 downregulated) in Pfn1 KO relative to control EC (Fig. 7A; Table S1 lists the top 50 up- and downregulated genes). Based on the absolute fold change in gene expression, Pfn1 and Ifi213 (an interferon-activated gene) ranked as the top-most downregulated and upregulated gene, respectively, in Pfn1 KO relative to control cells. When specifically probed for expression changes in immunomodulatory factor–encoding genes (specifically interleukins, CXC and CC family chemokines, and colony-stimulating factors [CSFs]), G-CSF/CSF3, CXCL10, and CCL5 were found to be robustly elevated (indicated by asterisks in Fig. 7B) in Pfn1 KO EC which is in agreement with our serum and/or CM Luminex data. Furthermore, consistent with the proinflammatory consequence of endothelial Pfn1 KO in vivo, ingenuity pathway analyses (IPA) of DEGs revealed that the top-ranked biological pathways activated in EC upon depletion of Pfn1 are heavily biased toward immunological pathways. These include interferon signaling, activation of interferon regulatory factor (IRF) by cytosolic pattern recognition receptors, pathogen-induced cytokine storm signaling, hypercytokinemia and hyperchemokinemia, and macrophage classical activation signaling pathways (Supplementary Fig. S5), with STAT and IRF families of transcription factors at the signaling hub of the top IPA-generated network (Fig. S6). Upstream regulator analysis (URA) of EC Pfn1–responsive genes in IPA predicted inhibition and activation of a wide range of transcriptional regulators as shown in the form of a volcano plot (Fig. 7C; Table S2 shows the list of top 10 activated and inactivated transcriptional regulators). As per these analyses, transcription factors STAT1 and IRF7 (interferon regulatory factor 7) were predicted to be the most robustly activated transcription factors in EC under Pfn1-depleted condition (Fig. 7C). Interestingly, TRIM24 (tripartite motif 24—an inhibitor of STAT1 transcription (28)) was predicted to be the most inhibited transcription factor in Pfn1-depleted EC. STATs (STAT1 and STAT2) and IRFs (IRF7 and IRF9) were represented as transcriptionally upregulated genes in several major signaling pathways relevant to our context including cytokine storm signaling, macrophage classical activation pathway, interferon signaling, and activation of IRFs by cytosolic pattern recognition receptors (Fig. S7). As an experimental validation, we found that the total as well as the phosphorylated (activated) forms of both STAT1 and IRF7 are elevated in EC when Pfn1 expression is depleted (Fig. 7D). Overall, these results are consistent with a well-established role of IRF/STAT1 signaling in the induction of proinflammatory cytokine/chemokine genes and classical activation of macrophages (29).

## Discussion

**Fig. 8.**
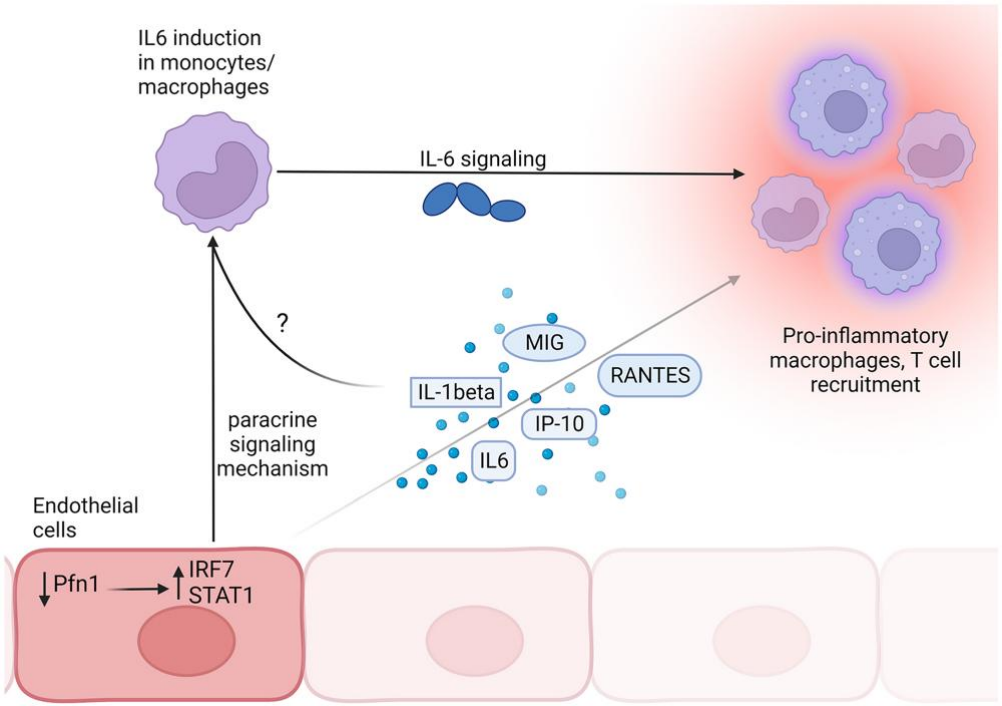
Schematic representation of how endothelial Pfn1 loss could potentially lead to a proinflammatory condition. We propose that loss of Pfn1 in EC directly stimulates select proinflammatory cytokine/chemokine expression/release through upregulating the intrinsic IRF7/STAT1 signaling in addition to potentiating proinflammatory gene expression in monocytes/macrophages in a paracrine signaling manner.

In this study, we report three novel findings. *First*, the induction of loss of EC Pfn1 in adult mammals leads to severe multiorgan pathology ultimately compromising survival, demonstrating that endothelial Pfn1 function is indispensable for life. These results contrast the characteristics of all other previously reported conditional Pfn1 KO mouse models where complete loss of Pfn1 function in various other types of differentiated cells resulted in discernible phenotypes but was tolerable. These results should be informative for the scientific community when choosing an appropriate mouse model for studies that pertain to the investigation into the role of endothelial Pfn1 in cardiovascular diseases and other chronic pathologies. *Second*, loss of endothelial Pfn1 leads to an inflammatory response marked by pronounced elevation of a number of major proinflammatory cytokines causing enrichment of myeloid-derived immune cells with proinflammatory characteristics. These data demonstrate for the first time that perturbation of Pfn1 expression in EC has dramatic immunological consequences. *Third*, loss of Pfn1 not only stimulates endothelial–intrinsic transcription/release of proinflammatory factors (consistent with the signature of increased IRF7 and STAT1 activation) but also potentiates endothelial-directed proinflammatory gene induction in macrophages in a paracrine manner thereby amplifying the inflammatory milieu, as schematized in Fig. 8.

The salient pathological features of Pfn1^EC-KO^ mice at the organ level found in this study are disrupted vasculature, stunted growth, tissue infarct, fibrosis, and immune cell infiltration. Disrupted vasculature in organs (kidney and liver) in Pfn1^EC-KO^ mice is consistent with endothelial Pfn1 dependency for angiogenesis previously shown by our group in early postnatal developmental setting (24) and, more recently, in a tumor angiogenesis setting in a renal cancer model (26). In the latter study, we further identified proangiogenic factor serpinE1 to be most heavily downregulated in the tumor microenvironment in response to loss of endothelial Pfn1. Prominent tissue infarct is suggestive of severe defect in circulation, which are expected to lead to some of the other observed histological changes including fibrosis and mononuclear immune cell infiltration. One of the striking features of Pfn1^EC-KO^ mice is abnormal peritoneal fluid accumulation and abdominal/thorax hemorrhage, the most likely cause of which is increased vascular permeability. Endothelial barrier function is critically dependent on the integrity of actin cytoskeleton and an intact tethering of adherens and tight junctions to the underlying cytoskeleton. A previous study showed that loss of the Ena/VASP family of F-actin-elongating proteins leads to compromised cell–cell junctions, endothelial barrier dysfunction, and hemorrhage in mice (30), similar to the phenotypes we observed in Pfn1^EC-KO^ mice. Ena/VASP proteins bind to Pfn1, and Pfn1's interaction augments actin polymerization ability of Ena/VASP (31). Therefore, it is possible that vascular leakiness in Pfn1^EC-KO^ mice could be partly attributed to cytoskeletal defects. Interestingly, we previously showed that acute silencing of Pfn1 expression in vascular EC stabilizes adherens and tight junctions conferring protection to VEGF-induced junctional disruption (5). Another group also reported improved retinal vascular barrier function in mice when subjected to intravitreal administration of Pfn1-shRNA–encoding adenovirus (32). We attribute the apparent contradictions between those results and the in vivo phenotype of Pfn1^EC-KO^ mice to at least two possible reasons. First, gene silencing of Pfn1 in EC reduces but does not completely eliminate Pfn1 expression and importantly does not cause cell death, unlike the effect of the total absence of Pfn1 expression. Second, we saw a robust systemic elevation of VEGF (a key vascular permeability–inducing factor) in Pfn1^EC-KO^ mice. Therefore, VEGF upregulation (compounded by apoptotic cell death) could be another major driving force for inducing vascular leakiness in Pfn1^EC-KO^ mice.

A key question that emerges from our study is whether the proinflammatory consequence of loss of endothelial Pfn1 is specific to some unique functional aspects of Pfn1 or a generic feature expected to result from the loss of any other key actin polymerization regulatory proteins. Disruption of endothelial barrier function contributing to local inflammation and cell death could certainly be a common phenotypic consequence of disruption of key regulators of actin polymerization. However, our cell culture–based studies showed that loss of endothelial Pfn1 leads to upregulation of proinflammatory factors not only in an EC-intrinsic manner but also in macrophages through a paracrine mechanism. Furthermore, our RNAseq studies revealed that many of the biological pathways that are predicted to be activated in EC upon Pfn1 depletion are related to immunological functions. Therefore, we reason that there are additional cellular consequences capable of impacting immune response that are unique to the loss of Pfn1.

The most prominent immunological consequence of endothelial Pfn1 depletion is the expansion of the myeloid-derived population of immune cells that are associated with proinflammatory characteristics. This finding is consistent with the systemic elevation of a number of proinflammatory cytokines in Pfn1^EC-KO^ mice. In the context of these data, it is interesting to note that in atherosclerosis setting, global heterozygous deletion of the Pfn1 gene reduces inflammation and macrophage activity conferring partial protection from the severity of atherosclerosis (9). While those data may appear to be contradictory to our findings, a direct comparison cannot be made between the two studies for at least three reasons. First, global heterozygous KO mice have attenuation (but not complete loss) of Pfn1 expression in all cell types, and therefore, it is difficult to attribute the immunological consequence of Pfn1 heterozygous mice solely to endothelial diminution of Pfn1 expression. Second, there is prior evidence for altered immune cell behavior (at least for cytotoxic T-cells) in response to perturbation of Pfn1 expression (33). Third, cell death and necrosis, features of Pfn1^EC-KO^ but not of the global Pfn1 heterozygous KO mouse model, could be the natural triggers of an inflammatory response in vivo.

Inflammatory macrophages with the M1-like phenotype are characterized by high expressions of IL1β and IL6 (34). A recent study also demonstrates the role of IL1β signaling in transcriptional upregulation of VEGF in macrophages (35). Macrophage-secreted IL6 can also stimulate the production of CXCL10 (a potent chemokine to recruit various immune cells including macrophages and T cells) in an autocrine manner which is indicative of a positive feedback loop between these cytokine signaling (36). Our cytokine profiling data show elevated levels of IL1β, IL6, and VEGF and CXCL10 (also a prominent M1-macrophage–associated marker) in Pfn1^EC-KO^ mice, with in vitro studies providing supportive evidence for both EC and macrophages being potential sources of these factors. STAT1 and IRF7 are key mediators of type I interferon response, regulated in a positive feedback loop controlling each other's expression, and major transcriptional regulators of several proinflammatory factors (e.g. CXCL10, CCL5, and CXCL9) that are elevated either in vitro or in vivo upon loss of endothelial Pfn1 (37, 38). Therefore, increased STAT1/IRF7 activation could be a potential molecular basis for proinflammatory gene induction in EC triggered by loss of Pfn1. Interestingly, we recently reported suppression of STAT1 expression in a tumor microenvironment when Pfn1 is overexpressed in EC (26). Although in that study, we did not discern which cell type reflects Pfn1-induced suppression of STAT1 expression in vivo, elevated STAT1 expression in Pfn1-KO EC as demonstrated herein consolidates our recent findings. How loss of Pfn1 increases STAT1/IRF7 expression/activation still remains to be elucidated. Future studies are needed to explore whether TRIM24 inactivation, DNA damage/cGAS-STING activation, and/or Toll-like receptor signaling by certain damage-associated molecular patterns are responsible for hyperactivation of IRF-STAT signaling in EC when depleted of Pfn1.

## Materials and methods

### Cell culture

Generation and culture conditions of hTERT-immortalized mouse EC bearing floxed-Pfn1 alleles have been described recently (26). For Pfn1 gene deletion, ECs were infected with either Ad-GFP (control) or Ad-Cre (also has a GFP reporter: Vector Biolabs, Malvern PA) at 400 MOI. Media were changed 24 h after infection, and ECs were used for experiments 5 to 6 days after initial infection. ECs were serum starved for 24 h prior to CM collection. For primary BMDM isolation, long bones of FVB mice were centrifuged at >8,000*g* for 3 min, and collected bone marrow was filtered and plated overnight. Nonadherent cells were collected the next day and stimulated with 10 ng/ml MCSF for 7 days to produce M0 macrophages. After 7 days, macrophages were replated and exposed to the CM derived from EC with or without additional stimulation of 100 ng/ml IFNγ (PeproTech, Cranbury NJ) for 24 h.

### Mouse studies

Cdh5-Cre-ERT2:Pfn1^flox/flox^ mice, as described in our previous study (20), were backcrossed into a BALB/C background for six generations prior to experimental studies. Pfn1 gene excision was performed by intraperitoneal injection of 100 μl TMX (dissolved in peanut oil at a concentration of 10 mg/ml) daily over a course of 5 days in 5- to 6-week-old mice. Details of genotyping primers to confirm floxed alleles of Pfn1, Cre, and Cre-mediated excision of Pfn1 are described in (31). Animals were euthanized for whole blood isolation by cardiac puncture and harvesting of various organs of interest (lung, liver, kidney, heart, and spleen). All animal experiments were conducted in compliance with an approved IACUC protocol according to the University of Pittsburgh Division of Laboratory Animal Resources guidelines.

### Cytokine analyses

Mouse serum (prepared from whole blood and further diluted with PBS at a 1:1 ratio) and mouse EC CM were probed for cytokine/chemokine expression levels by Luminex analysis by service provided by Eve Technologies (Calgary, AB, Canada) using the MD32 (32-analyte murine discovery panel) panel. For mouse serum analyses, analyte values were normalized to the average of wild-type (WT) animals within litters. For CM analyses, analyte values were normalized to the corresponding average WT CM values per analyte basis.

### Flow cytometry analyses

Whole blood or spleen was passed through a 70-μm cell strainer, treated with 1× RBC lysis buffer to reduce red blood cell contamination, and incubated with Brilliant Stain Buffer (BD Biosciences, San Jose, CA) to prevent signal overlap between blue and ultraviolet markers. Samples were stained with either a full panel of immune cell–specific antibodies or buffer only for 30 min at 4°C, spun, and fixed in fixation/permeabilization buffer overnight, followed by next-day intracellular factor staining for 30 min at room temperature (see Supplementary Table S3 for immune panel antibody and the respective dilution information). Samples were run on a Fortessa FACS Aria II, and the data were analyzed by FlowJo (FlowJo Inc., Ashland, OR) software.

### Immunohistochemistry and TEM analyses

For CD31 immunostaining, paraffin-embedded tissue sections were deparaffinized by heating to 60°C for 20 min, cleared via xylene, and dehydrated in 100% ethanol. Sections were blocked for 10 min using Protein Block included in a rabbit-specific HRP/DAB (ABC) Detection IHC kit (Abcam, ab64261), and slides were incubated overnight at 4°C with primary anti-CD31 antibody (clone D8V9E, catalog no. 77699, Abcam, dilution 1:100). This was followed by incubation with a biotin-labeled anti-rabbit secondary antibody for 10 min, washing with PBS-Tween, and incubation with streptavidin–peroxidase for 10 min. The staining was then detected with DAB substrate by incubation for 5–6 min. Slides were counter-stained with hematoxylin for 2 min before dehydration, mounting, and imaging.

For H&E staining, the same initial steps were adopted before incubation of histosections with Mayer's hematoxylin (Lillie's modification) for 2 to 3 min. Slides were washed in deionized water, color adjusted with 5% glacial acetic acid, incubated with bluing reagent for 10–15 s, washed in DI, and stained with eosin for 2–3 min before dehydration and mounting. For apoptotic cell analysis, Click-IT TUNEL colorimetric kit (Invitrogen, Waltham, MA) staining was performed according to the manufacturer-supplied protocol. Masson's trichrome staining was performed by the McGowan Institute of Regenerative Medicine Histology Core at the University of Pittsburgh.

For TEM, tissues were harvested and immersion fixed in 2.5% glutaraldehyde and 2% paraformaldehyde in PBS overnight at 4°C. Following fixation, tissues were washed 3× in PBS and then postfixed in aqueous 1% OsO_4_ and 1% K_3_Fe(CN)_6_ for 1 h. Following three PBS washes, the tissue was dehydrated through a graded series of 30–100% ethanol and 100% propylene oxide and then infiltrated in 1:1 mixture of propylene oxide:Polybed 812 epoxy resin (Polysciences, Warrington, PA) for 1 h. After several changes of 100% resin over 24 h, tissue was embedded in molds and cured at 37°C overnight, followed by additional hardening at 65°C for 2 more days. Ultrathin (60 nm) cross-sections of the liver or kidney were collected on copper grids and stained with 1% uranyl acetate for 10 min, followed by 1% lead citrate for 7 min. Sections were imaged using a JEOL JEM 1400 Flash transmission electron microscope (Peabody, MA) at 80 kV with a bottom-mount AMT 2k digital camera (Advanced Microscopy Techniques, Danvers, MA).

### Immunoblot

Total cell lysate (TCL) was collected using a modified RIPA buffer (25 mM Tris-HCl, pH 7.5, 150 mM NaCl, 1% [v/v] Nonidet P-40, 5% [v/v] glycerol), 1 mM EDTA, 50 mM NaF, 1 mM sodium pervanadate, along with 6× sample buffer with SDS diluted to 2% in final buffer. TCL was run on an SDS–PAGE and immunoblotted using antibodies specific for Pfn1 (Abcam, ab124904; 1:3,000), IRF7 (Cell Signaling Technology, Danvers MA, 72073; 1:1,000), p-IRF7 (Cell Signaling Technology, 24129; 1:1,000), STAT1 (Cell Signaling Technology, 9172; 1:1000), p-STAT1 (Cell Signaling Technology, 9167; 1:1,000), and GAPDH (Sigma-Aldrich, G9545; 1:2,000).

### Gene expression analyses

For assessing Pfn1 expression in ECs in vivo, single cell isolate was collected by collagenase-mediated kidney digestion and labeled with MACS mouse CD31 magnetic beads (130-097-418, Miltenyi Biotec; Gaithersburg MD) prior to isolation using magnetic LS columns according to manufacturer's recommended protocol. Unlabeled effluent was collected as a negative control. To assess gene expression in circulating myeloid populations, whole blood was labeled with MACS mouse F4/80 beads (130-110-443, Miltenyi Biotec) and isolated using LS columns. Cellular RNA was extracted using the Qiagen RNeasy Mini Kit (Qiagen, Valencia, CA) as per the manufacturer's protocol. Complementary DNA (cDNA) synthesis from the extracted RNA was then performed using the Qiagen QuantiTect Reverse Transcription kit (Qiagen, Germantown MD) using the Applied Biosystems Veriti Thermal Cycler (Applied Biosystems, Waltham MA) following the manufacturer's instructions. Collected cDNA was amplified using SYBR Green Gene Expression Master Mix on the StepOnePlus real-time PCR system (Applied Biosystems) using primers for specific genes of interest. For product amplification, the plate was ramped to 50°C for 4 min, followed by 2 min at 95°C preamplification cycling. Cycling conditions of 95°C for 20 s, 49.7°C for 25 s, and 95°C for 50 s were used for 60 cycles (see Supplementary Table S4 for information on primer sequences).

### RNAseq

Total RNA was submitted to Azenta Genewiz (Burlington, MA) for quality control analyses, preparation of multiplexed paired-end libraries, polyA selection, and sequencing on an Illumina platform with 20 to 30 million reads per sample. FASTQ data processing and downstream analyses are described in detail in our recent study (26).

### Statistics

All statistical tests were performed using GraphPad Prism 9 software. For experiments with a small sample size and nonnormal distribution of data, nonparametric Mann–Whitney *U* test was used for statistical comparison of data between the groups. For experiments involving a larger sample size and normal sample distribution, an unpaired *t*-test was used. A *P*-value less than 0.05 was considered to be statistically significant.

## Supplementary Material

pgad305_Supplementary_Data

## Data Availability

All data are included in the article and/or supporting information.
