# Supplementary material for: Vascular endothelial cell-specific disruption of the profilin1 gene leads to severe multiorgan pathology and inflammation causing mortality
Source: PNAS Nexus. 2023 Sep 16;2(10):pgad305. doi: 10.1093/pnasnexus/pgad305 (PMC10541205; doi:10.1093/pnasnexus/pgad305)
Supplement: pgad305_Supplementary_Data [file pgad305_supplementary_data.zip › PNASNEXUS-PNASNEXUS-2022-01285R-s04.docx]

**TABLE S3**

| **Antigen** | **Fluorophore** | **Dilution** | **Catalog** | **Cat#** |
| --- | --- | --- | --- | --- |
| CD45.2 | R718 | 1:50 | BD Biosciences | 567585 |
| MHC Class II (IA/IE) | BV510 | 1:200 | BD Biosciences | 743871 |
| CD11b | BV421 | 1:400 | BD Biosciences | 562605 |
| F4/80 | APC-Cy7 | 1:25 | Biolegend | 123118 |
| Ly6G | BV786 | 1:200 | BD Biosciences | 740953 |
| CD19 | BUV395 | 1:100 | BD Biosciences | 563557 |
| CD11c | BV650 | 1:100 | BD Biosciences | 564079 |
| Ly6C | PE/Cy7 | 1:100 | BD Biosciences | 560593 |
| CD8 | FITC | 1:200 | BD Biosciences | 553030 |
| CD3 | APC | 1:100 | BD Biosciences | 565643 |
| FOXP3 | PE | 1:50 | BD Biosciences | 560408 |
| CD4 | PerCP | 1:100 | BD Biosciences | 553052 |

**Table S3:** Details of antibodies used for Flow Cytometry Analyses
