# Supplementary material for: Vascular endothelial cell-specific disruption of the profilin1 gene leads to severe multiorgan pathology and inflammation causing mortality
Source: PNAS Nexus. 2023 Sep 16;2(10):pgad305. doi: 10.1093/pnasnexus/pgad305 (PMC10541205; doi:10.1093/pnasnexus/pgad305)
Supplement: pgad305_Supplementary_Data [file pgad305_supplementary_data.zip › PNASNEXUS-PNASNEXUS-2022-01285R-s05.docx]

**TABLE S4**

| Gene | Primer sequence | Amplicon size |
| --- | --- | --- |
| IL6 | F: 5’— CTG CAA GAG ACT TCC ATC CAG—3’  R: 5’— AGT GGT ATA GAC AGG TCT GTT GG—3’ | 131 |
| Pfn1 | F: 5’—CGA GAG CAG CCC CAG TAG CAG C—3’  R: 5’—ACC AGG ACA CCC ACC TCA GCT G—3’ | 180 |
| 18S | F: 5’—GCA ATT ATT CCC CAT GAA CG—3’  R: 5’—GGC CTC ACT AAA CCA TCC AA—3’ | 123 |
| GAPDH | F: 5’ – CGG AGT CAA CGG ATT TGG TCG TAT – 3’  R: 5’ – AGG CTT CTC CAT GGT GGT GAA GAC – 3’ | 307 |

**Table S4:** Primer sequences for qRT-PCR analyses of genes of interest.
