# Supplementary material for: Vascular endothelial cell-specific disruption of the profilin1 gene leads to severe multiorgan pathology and inflammation causing mortality
Source: PNAS Nexus. 2023 Sep 16;2(10):pgad305. doi: 10.1093/pnasnexus/pgad305 (PMC10541205; doi:10.1093/pnasnexus/pgad305)
Supplement: pgad305_Supplementary_Data [file pgad305_supplementary_data.zip › PNASNEXUS-PNASNEXUS-2022-01285R-s01.pdf]

Supplementary Information for

Vascular endothelial cell-specific disruption of the *profilin1* gene leads to severe multi-organ pathology and inflammation causing mortality

Abigail Allen-Gondringer,[1] David Gau[1], Christopher Varghese[1], David Boone[2], Donna Stolz,[3] Adriana Larregina,[4,5] Partha Roy[1,6]

[1] Bioengineering, [2]Biomedical Informatics, [3] Cell Biology, [3]Dermatology, [4]Immunology, [5]Pathology, University of Pittsburgh, Pittsburgh, PA

Correspondence: Partha Roy, 306 CNBIO, 300 Technology Drive, Pittsburgh, PA 15219

Email: par19@pitt.edu

This PDF file includes:

Figures S1 to S7 (with legends)

Other supplementary materials for this manuscript include the following:

Table S1: A list of top differentially expressed genes in EC in response to Pfn1 gene KO

Table S2: A list of IPA-predicted top upstream transcriptional regulators activated and inactivated in EC in response to Pfn1 gene KO

Table S3: Details of antibodies used in flow cytometry analyses

Table S4: Details of RT-PCR primers

Allen-Gondringer et al. Fig S1

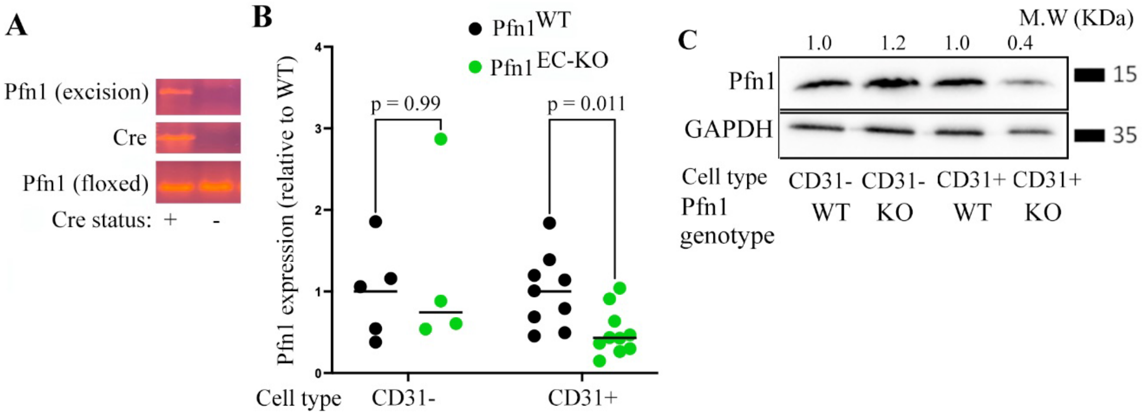

Fig. S1: A) Genotyping PCRs to confirm the presence of floxed (340 bp) alleles of Pfn1, Cre and Cre-mediated 700 bp Pfn1 excision band in Cre-positive mice. **B)** Quantitative PCR-based evaluation of Pfn1 gene expression in CD31+ vs and CD31- (effluent sorted) single cell isolates from the kidney of Pfn1$^{EC\text{-}KO}$ mice relative to those from Pfn1^{WT} mice. Each data point represents a single mouse. **C)** Immunoblot-based validation of downregulation of Pfn1 expression at the protein level in CD31+ but not in CD31- kidney cell isolates in Pfn1$^{EC\text{-}KO}$ mice (GAPDH blot serves as the loading control). The numbers indicate GAPDH-normalized Pfn1 band intensity in KO relative to the WT group for a given cell type.

Allen-Gondringer et al. Fig S2

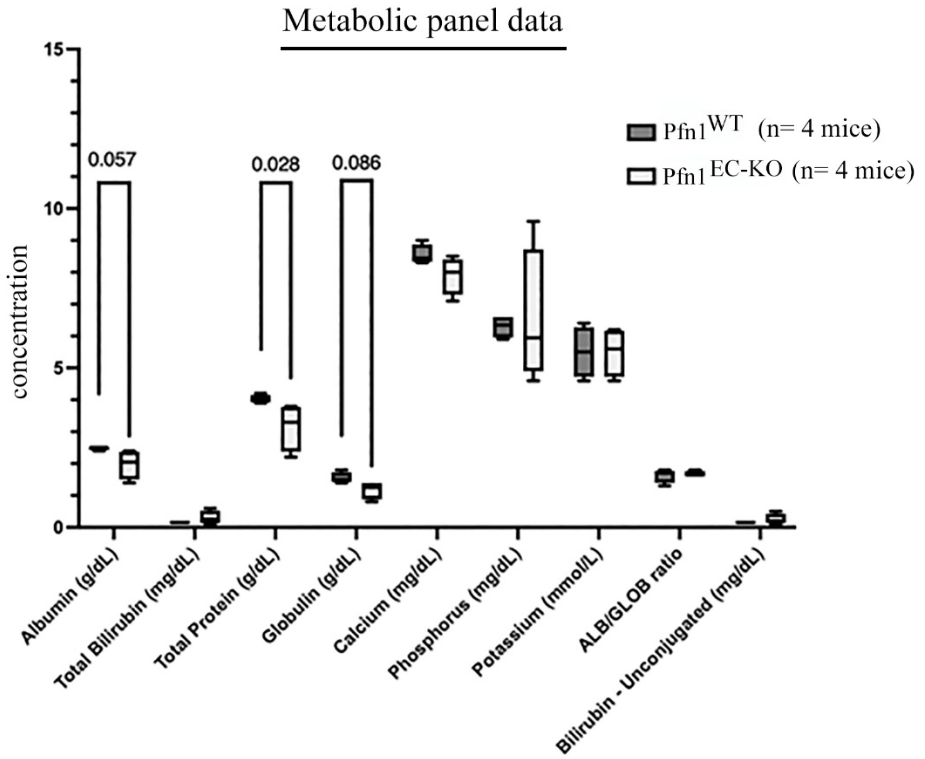

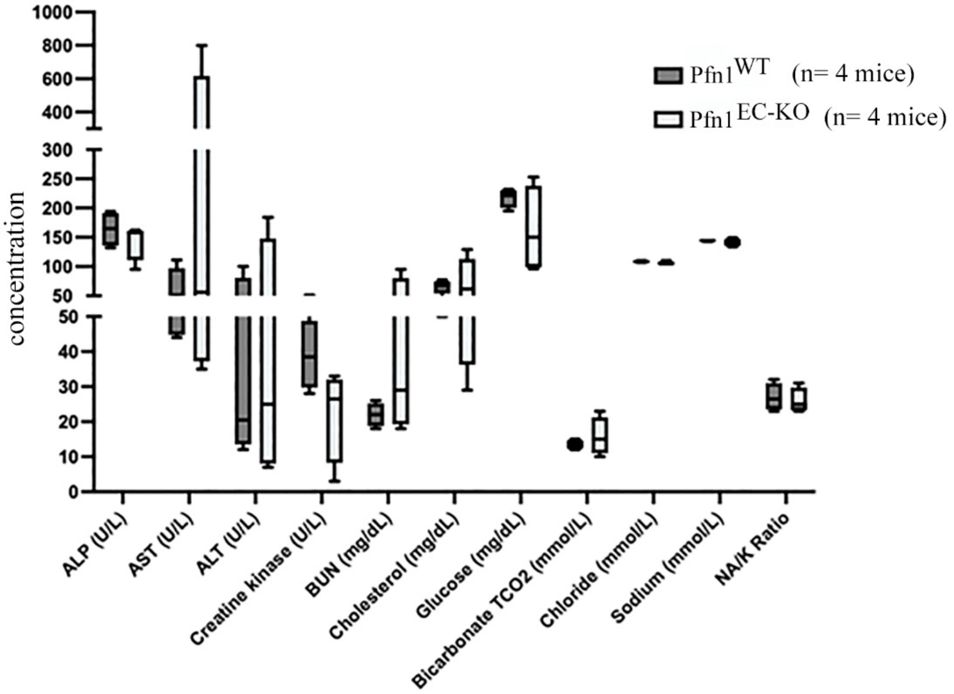

Fig S2: Relative abundance of various serum analytes of $Pfn1^{WT}$ and $Pfn1^{EC-KO}$ mice based on the results of Idexx serum metabolic panel analyses.

Allen-Gondringer et al. Fig S3

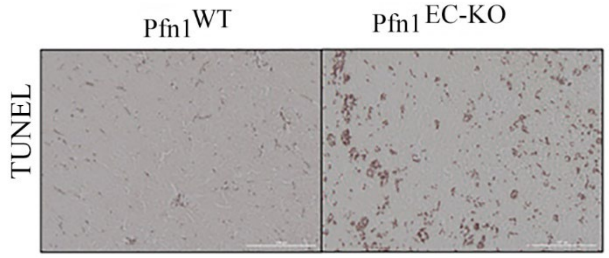

Fig S3: TUNEL staining of day 18 liver tissue sections show extensive apoptotic cell death in Pfn1$^{EC\text{-}KO}$ liver (Scale bar – 100 µm)

Allen-Gondringer et al. Fig S4

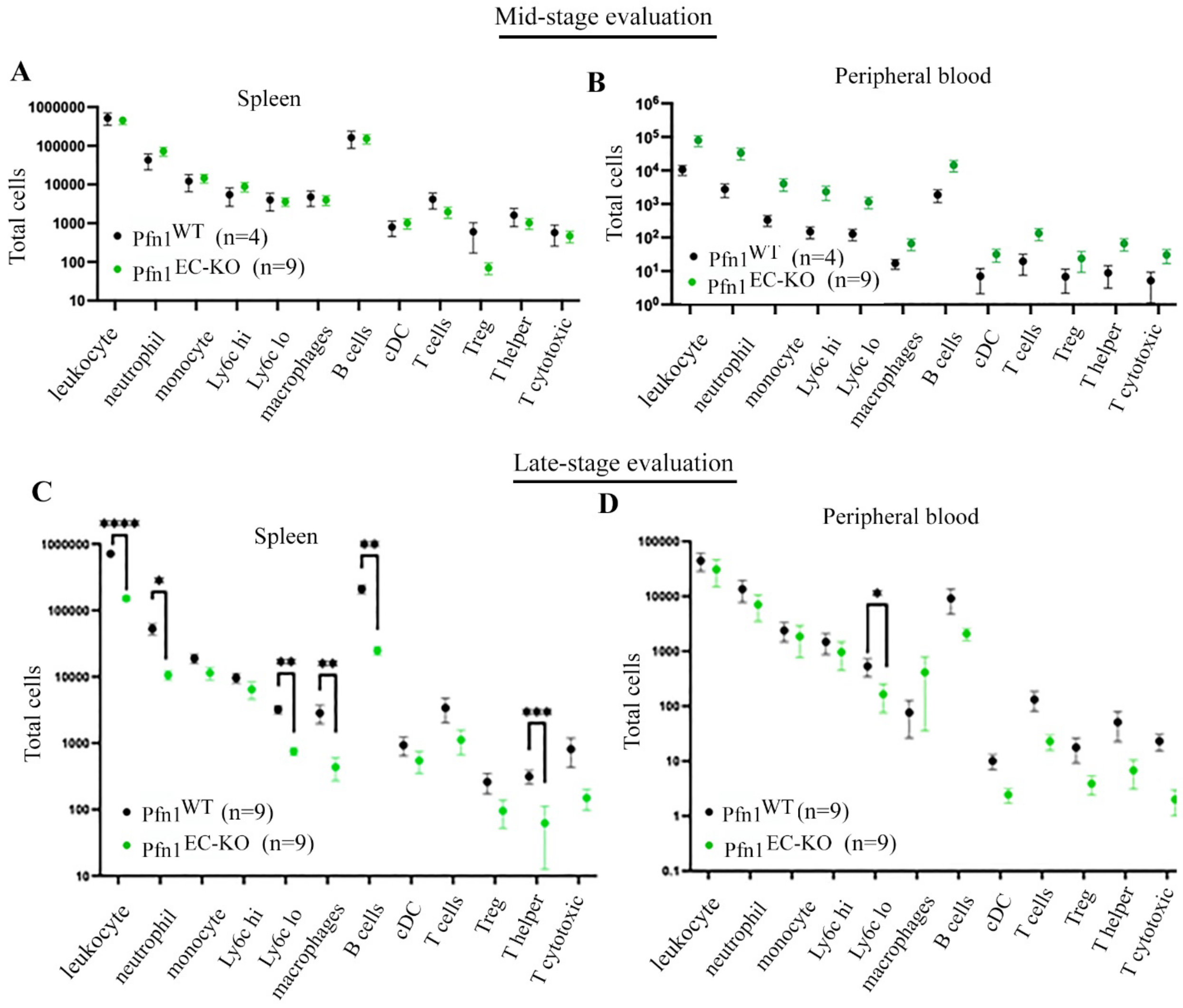

Fig S4: Flow cytometry-based assessment of total count of leukocytes and various immune cell subpopulations in the spleen and peripheral blood of Pfn1^{WT} vs. Pfn1$^{EC\text{-}KO}$ mice at the indicated time-points ('n' indicates the number of animals in each group).

Allen-Gondringer et al. Fig S5

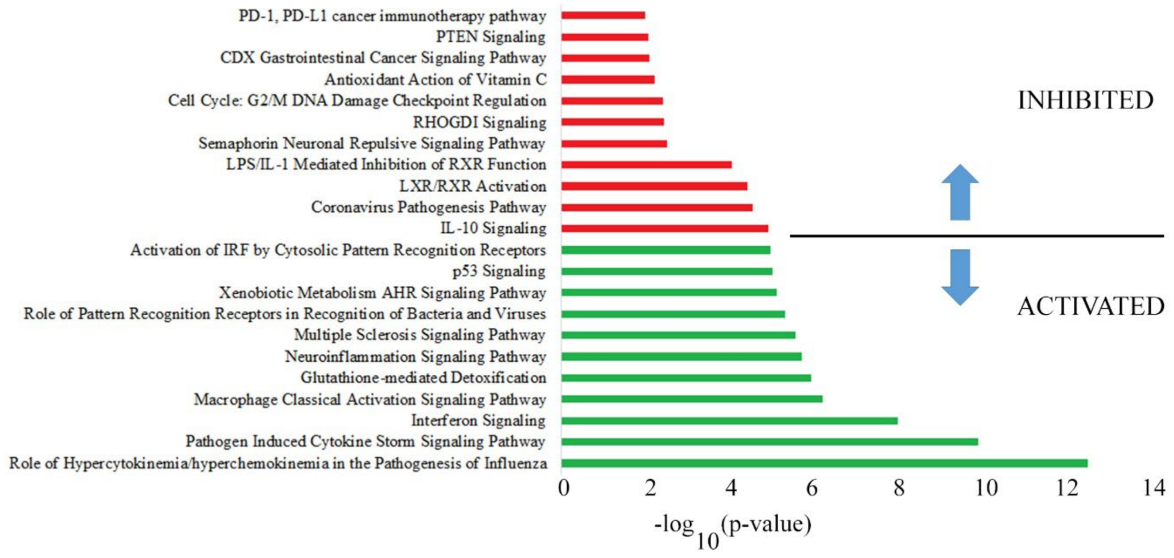

Fig S5: IPA-based prediction of top pathways up- and down-regulated in Ad-Cre- relative to Ad-GFP-infected EC.

Allen-Gondringer et al. Fig S6

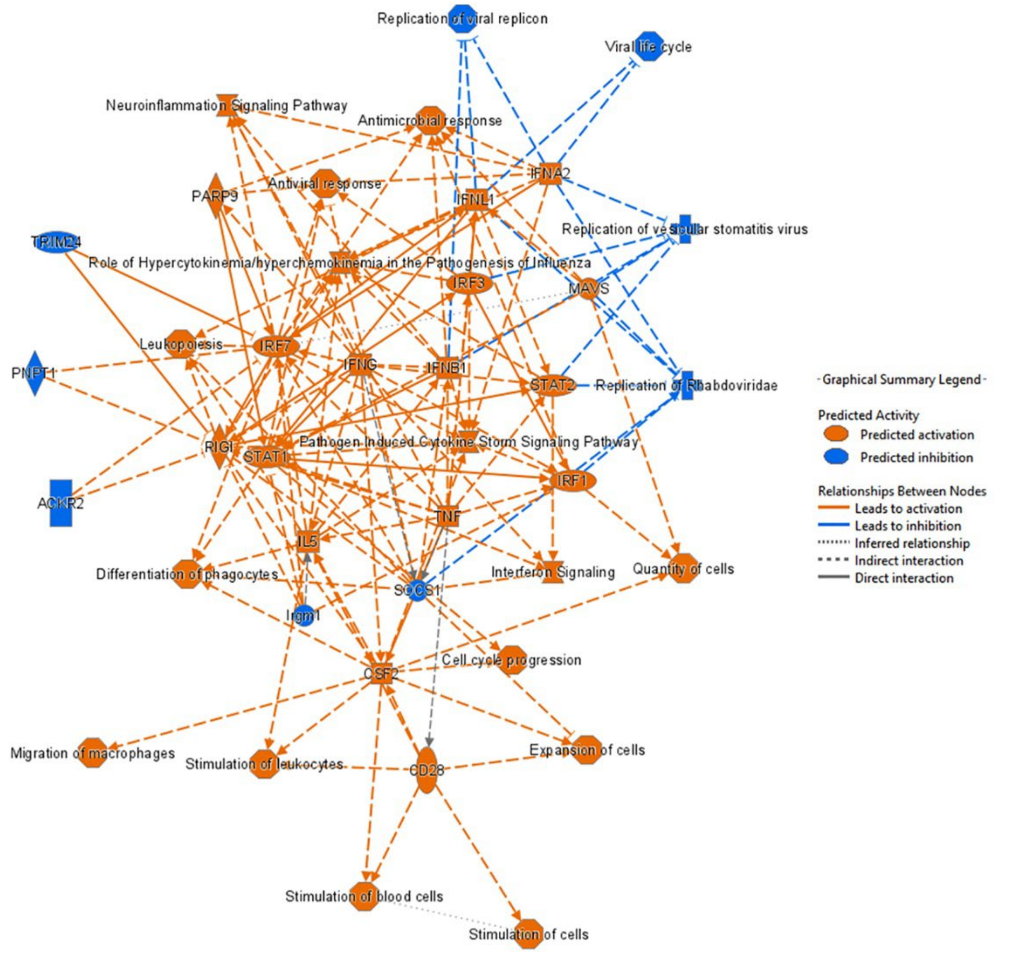

Fig S6: Top IPA signaling network showing STAT and IRFs as major signaling hubs linking to top biological pathways impacted by Pfn1 gene KO in EC.

Allen-Gondringer et al. Fig S7

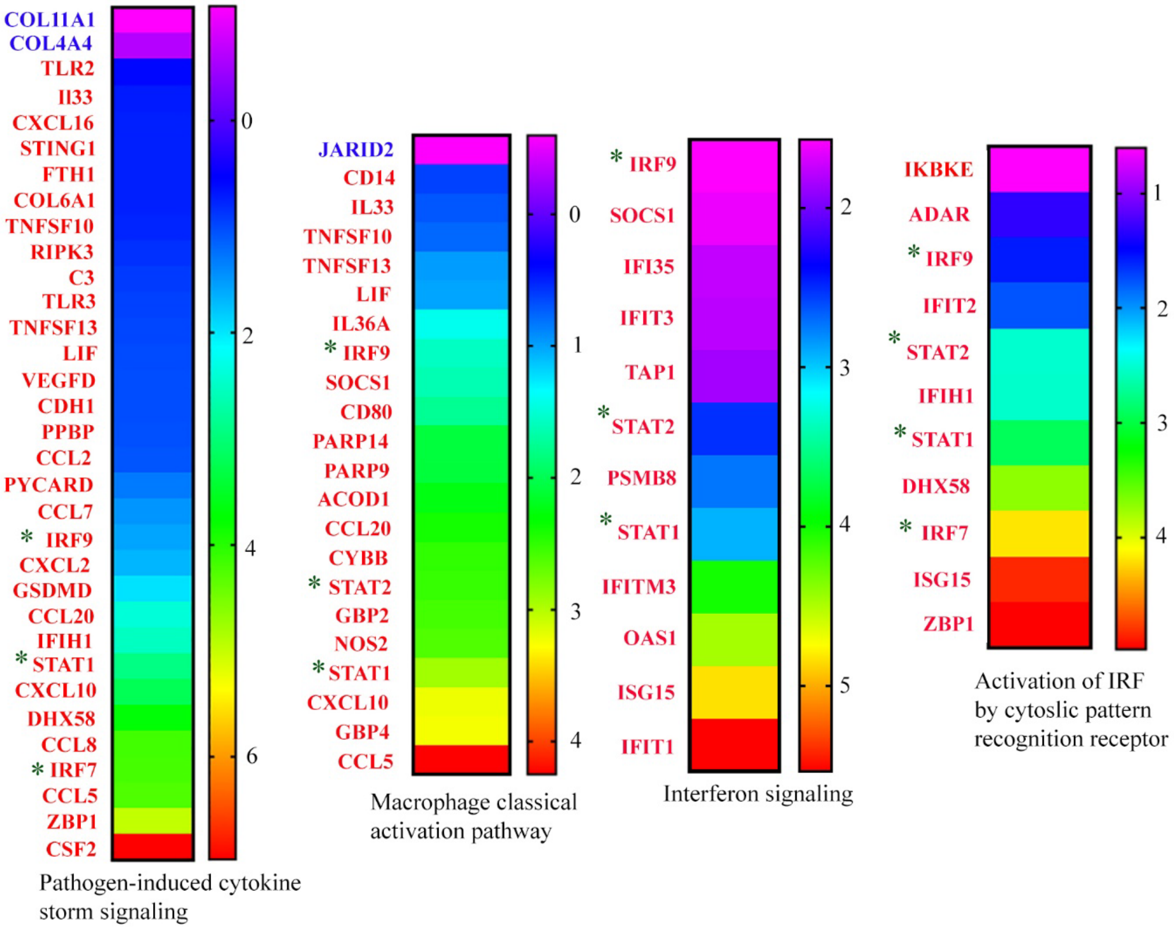

Fig S7: Heat plots showing the average log2 fold-change of differentially expressed genes in the indicated pathways in response to Pfn1 gene knockout in EC (genes indicated by blue and red are transcriptionally down- and up-regulated, respectively).
